# The Role of the Periaqueductal Gray in the Modulation of Pain in Males and Females: Are the Anatomy and Physiology Really that Different?

**DOI:** 10.1155/2009/462879

**Published:** 2009-01-28

**Authors:** Dayna R. Loyd, Anne Z. Murphy

**Affiliations:** Neuroscience Institute, Center for Behavioral Neuroscience, Georgia State University, P.O. Box 4010, Atlanta, GA 30302, USA

## Abstract

Anatomical and physiological studies conducted in the 1960s identified the periaqueductal gray (PAG) and its descending projections to the rostral ventromedial medulla (RVM) and spinal cord dorsal horn, as a primary anatomical pathway mediating opioid-based analgesia. Since these initial studies, the PAG-RVM-spinal cord pathway has been characterized anatomically and physiologically in a wide range of vertebrate species. Remarkably, the majority of these studies were conducted exclusively in males with the implicit assumption that the anatomy and physiology of this circuit were the same in females; however, this is not the case. It is well established that morphine administration produces greater antinociception in males compared to females. Recent studies indicate that the PAG-RVM pathway contributes to the sexually dimorphic actions of morphine. This manuscript will review our anatomical, physiological, and behavioral data identifying sex differences in the PAG-RVM pathway, focusing on its role in pain modulation and morphine analgesia.

## 1. Introduction

**Figure 1:**
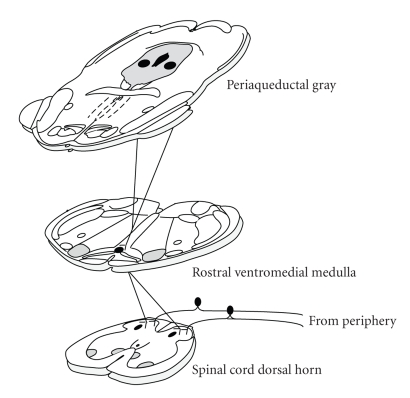
A schematic of the descending inhibitory pathway for pain modulation illustrating the projections from the midbrain periaqueductal gray to the brainstem RVM and the spinal cord dorsal horn.

It was first reported that electrical stimulation of the midbrain periaqueductal gray (PAG) produced profound analgesia in the male rat in 1969 [1]. Since then, the anatomical and physiological organization of the PAG and its descending projections to the rostral ventromedial medulla (RVM) and dorsal horn of the spinal cord have been well characterized in a variety of species, including the rat [2–9], cat [10–18], primate [19, 20], and rabbit [21] (see Figure 1). The PAG-RVM-spinal cord pathway comprises an essential neural circuit for opioid-based antinociception [6, 18, 22]. Intra-PAG administration of the mu opioid receptor (MOR) agonist morphine, the most commonly prescribed opiate for persistent pain relief, produces naloxone-reversible analgesia [23] as well as naloxone-reversible excitation of RVM neurons [7, 24, 25]. Similarly, lesions of the PAG or intra-PAG administration of MOR antagonists [26–29] attenuate the antinociceptive effects of systemic morphine across a wide range of analgesiometric tests [30]. Studies utilizing autoradiography, immunohistochemistry, and in situ hybridization have shown that the PAG contains a high density of MOR [31–38], with approximately 27–50% of PAG neurons retrogradely labeled from the RVM expressing MOR [35, 37].

While it is well established that the PAG-RVM-spinal cord pathway is essential for the analgesic actions of both systemic and intra-PAG morphine, these early studies were conducted exclusively in male subjects. Only recently have studies begun including “sex” as an independent variable, and it is becoming increasingly clear that morphine does not produce the same degree of antinociception in males and females, especially following the induction of persistent pain. Sex differences in morphine potency were first reported in rodents in the late 1980s, when it was shown that systemic morphine administration produced a significantly greater degree of antinociception in males using acute pain assays [39–42]. This phenomenon has been repeated in multiple studies employing animal models of pain, including orofacial [43] and visceral [44, 45] pain models, as well as persistent somatic pain models [38, 46–52]. Although results on the contrary are also reported, generally these studies have shown that morphine produces a significantly greater degree of analgesia in males in comparison to females. Indeed, we have recently reported that male rats have a significantly higher MOR expression in the PAG, which is positively correlated with morphine analgesia in male but not female rats [38].

Recently, clinical studies in humans have also reported sex differences in morphine analgesia. Of the limited number of studies that examined “gender” or “sex” as an independent variable, it has been reported that males experience greater morphine analgesia compared to females [53–55]. In fact, one study reported that females required 30% more morphine to reach the same level of analgesia as males [55]. Similar to the rodent literature, the results in human studies are not unequivocal. Sarton et al. [56] reported greater morphine analgesia in females, while two studies reported no sex difference [57, 58]. Sex differences in morphine consumption also have been reported [59]; however, given that the majority of negative side effects associated with morphine consumption, including nausea, headache, and dysphoria [57, 60], are exacerbated in females compared to males, morphine consumption is not a reliable indicator of morphine analgesia.

Sex differences in opioid analgesia are not limited to mu opioid agonists. In both human and animal studies, sex differences in the analgesic effects of kappa or delta opioid agonists have also been reported, although again, not without controversy [61–65]. Several factors are likely to contribute to the disparate results between studies reporting the presence or absence of a sex difference in opioid analgesia, including differences in the type of pain being examined (e.g., experimental acute pain versus postoperative pain versus a chronic pain state), the route of drug administration (e.g., oral versus intravenous versus intrathecal), the strain differences in the rodents studies, and the efficacy of the opiate being administered. Sex differences in basal pain sensitivity, as well as estrous cycle effects, may also contribute [54, 55, 57, 66–79].

While it is clear that sex differences in opioid analgesia are not a simple and straightforward phenomenon, when sex differences are reported, they are not trivial in magnitude. In our persistent inflammatory [38, 46] and visceral pain [44, 45] studies, the ED_50_ for females is twice the ED_50_ of males. Similarly, morphine is approximately 5-fold more potent in producing antihyperalgesia in arthritic males compared to arthritic females [52]. Sex differences in morphine analgesia are not due to sex differences in the pharmacokinetics of morphine in humans [56] or rodents [50]. Rather, sex differences in morphine analgesia are likely related to the inherent differences in how the central nervous system of males and females respond to opiates. To date, the mechanism(s) underlying the sexually dimorphic actions of morphine remain unknown.

Given that the PAG and its descending projections to the RVM and dorsal horn of the spinal cord provide a primary pathway for the actions of opiates in pain modulation, inherent differences in this pathway could contribute to the sexually dimorphic actions of morphine. Thus, we tested three hypotheses: (1) are there sex differences in the anatomical organization of the PAG-RVM pathway? (2) is there a sexually dimorphic response of the PAG-RVM output neurons to persistent pain? (3) does the administration of morphine differentially engage the PAG-RVM pathway in male and female rats?

## 2. Sexually Dimorphic Organization of a Descending Pain Inhibitory Pathway

**Figure 2:**
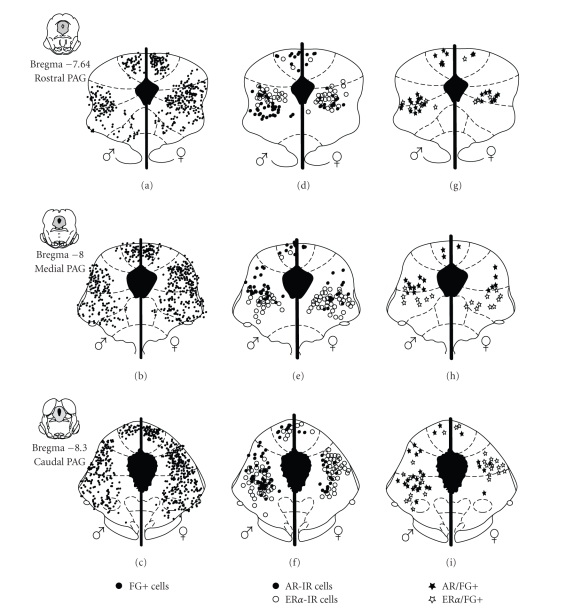
(a)–(c) Distribution of cells retrogradely labeled (FG+) from the RVM in males (left) and females (right) at three representative rostrocaudal levels of the periaqueductal gray. Each black circle represents one FG+ cell. (d)–(f) Distribution of PAG cells that were immunoreactive for AR (closed circles) or ER*α* (open circles). (g)–(i) Distribution of PAG cells retrogradely labeled from the RVM that were also immunoreactive for AR (closed stars) or ER*α* (open stars).

**Figure 3:**
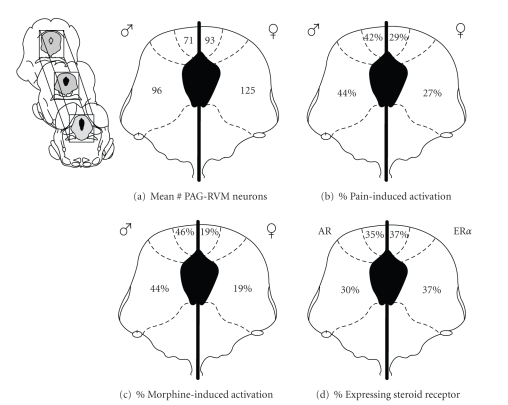
(a) Mean number of PAG cells retrogradely labeled from the RVM across the rostrocaudal axis in males (left) and females (right). (b) Percentage of Fos-positive neurons that were retrogradely labeled from the RVM in males (left) and females (right) following twenty-four hours of inflammation. (c) Average of the percentage of AR (left) and ER*α* (right) receptor-expressing PAG cells retrogradely labeled from the RVM. (d) Percentage of Fos-positive neurons that were retrogradely labeled from the RVM in males (left) and females (right) following twenty-four hours of inflammation and one hour of morphine (5 mg/kg).

We used neuroanatomical tract-tracing techniques to examine whether there were qualitative and/or quantitative differences in the neural projection from the PAG to the RVM in male and female rats. Consistent with previous anatomical studies [2, 80, 81], we reported that the dorsomedial, lateral and ventrolateral PAG heavily project to the RVM in both male and female rats [82]. While no qualitative sex differences were noted in the overall distribution of PAG-RVM projection neurons, females had significantly more PAG-RVM output neurons across the rostrocaudal axis of the PAG compared to males [83, 84] (Figures 2(a)–2(c)). The average number of retrogradely labeled cells across the rostrocaudal extent of the PAG was greater by a third in female compared to male rats (Figure 3(a)). The most prominent sex difference in retrograde labeling was observed within the lateral and ventrolateral regions of the PAG, an area known to contain a dense distribution of MOR [34, 37].

## 3. Sexually Dimorphic Response of the PAG-RVM Pathway to Persistent Inflammatory Pain

Inflammatory pain results in the activation of descending modulatory circuits [8, 85] and contributes to both hyperalgesia and antinociception [86–89]. We found that the persistent inflammatory pain induced by injection of complete Freund's adjuvant (CFA) into the rat hindpaw caused extensive activation of PAG neurons as measured by Fos labeling. Interestingly, this activation was comparable (both quantitatively and qualitatively) in male and female rats [82]. However, when the analysis was restricted to PAG neurons retrogradely labeled from the RVM, while females have almost twice the number of PAG-RVM output neurons in comparison to males, very few of these cells in female rats expressed inflammation-induced Fos, suggesting that this circuit is preferentially activated in males (Figure 3(b)). Indeed we found that, overall, persistent inflammatory pain activated approximately 43% of PAG-RVM neurons in the dorsomedial, lateral and ventrolateral PAG of males, but only half as many PAG-RVM output neurons were activated by inflammatory pain in females. Activation of the PAG and its descending outputs to the RVM results primarily in the inhibition of dorsal horn neuronal responses to acute noxious stimuli [90–95]; therefore, one would predict that given the greater activation of the circuit in males than females, males should have displayed reduced hyperalgesia following induction of plantar inflammation. However, in our behavioral studies, we found no sex differences in either baseline withdrawal latencies or in CFA-induced hyperalgesia. Therefore, our finding that the PAG-RVM descending circuit is not being engaged to the same degree by persistent inflammatory pain in males and females suggests that there is an alternative mechanism for endogenous pain modulation in female rats [96–99].

We have recently begun exploring this possibility using combinatorial anterograde and retrograde tract-tracing in combination with persistent pain-induced Fos labeling. The results of these studies suggest that there are indeed sex differences in both the efferent and afferent projections of the PAG. Specifically, the amygdala, ventromedial hypothalamus, and periventricular nucleus project more heavily to the PAG in females than males. In contrast, the medial preoptic area, parabrachial nucleus, and locus coeruleus project more heavily to the PAG in males than females [100]. In addition, our data indicate that the projections to the parabrachial nucleus, locus coeruleus, and the A5/A7 noradrenergic cell group appear to be greater in males (Loyd and Murphy, unpublished observations). Obviously, further research on the anatomy and physiology of pain modulatory circuits in females is warranted.

## 4. Sex Differences in the Activation of the Descending Inhibitory Pathway by Morphine

Although sex differences in PAG-RVM output neuron activation do not appear to contribute to sex differences in pain, they do appear to contribute to sex differences in morphine analgesia. Until recently, all studies examining the mechanisms of morphine action in the PAG were conducted exclusively in males; therefore it was unknown whether morphine administration has the same physiological effect on PAG neurons in females. Electrophysiological studies of PAG neurons are limited because they examine the response of a single neuron [7, 17, 101–109]. We have addressed this problem by using tract-tracing techniques and Fos labeling to measure the activity of *populations* of PAG-RVM neurons in the PAG of males and females.

**Figure 4:**
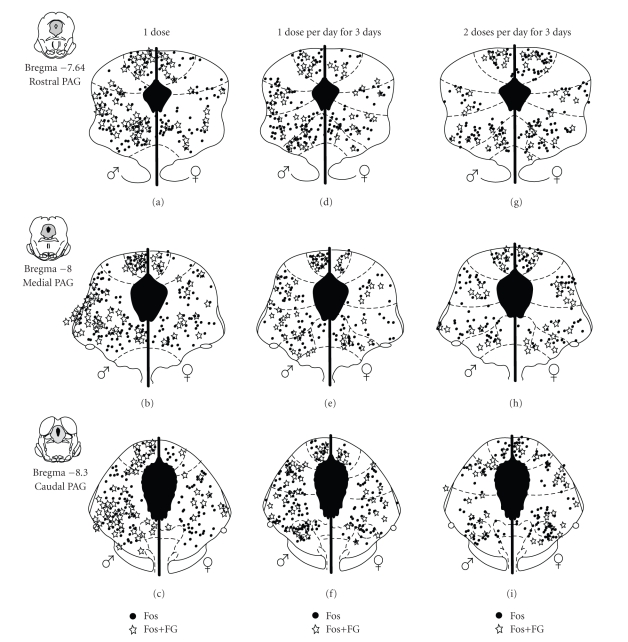
Distribution of PAG cells expressing Fos (black circles) and cells retrogradely labeled from the RVM expressing Fos (stars) following: (a)–(c) one 5 mg/kg dose of morphine; (d)–(f) one 5 mg/kg dose of morphine per day for three consecutive days; (g)–(i) or two 5 mg/kg doses of morphine per day for three consecutive days in males (left) and females (right) at three representative rostrocaudal levels of the PAG.

Systemic morphine administration attenuates the persistent pain-induced Fos expression within the PAG of male but not female rats [82] and is consistent with our data showing that the ED_50_ for systemic morphine is approximately twofold higher in females compared to males whether administered systemically [46] or directly into the PAG [38]. Interestingly, morphine administration, in the absence of pain, resulted in a twofold greater activation of PAG neurons compared to saline administration [84]. No sex difference was observed in the activation of PAG neurons by morphine (see the black circles in Figures 4(a)–4(c)), suggesting that in the absence of pain, morphine is equipotent in its ability to depolarize PAG neurons. However, when the analysis was limited to PAG neurons projecting to the RVM, the number of neurons activated by morphine was consistently and significantly higher in males compared to females (see the stars in Figures 4(a)–4(c)) [84]. Indeed, approximately half of PAG-RVM neurons in males were activated by morphine, whereas only 20% were activated in females (see Figure 3(c)). These results corroborate previous studies demonstrating that morphine results primarily in the net excitation of PAG-RVM neurons, most likely through the removal of tonic GABA inhibition [35, 104, 110, 111]. The finding that very few PAG-RVM neurons were activated by morphine in females suggests that morphine may be limited in effectiveness as a pain modulator.

Given that more PAG neurons project to the RVM in female compared to male rats, it is possible that pain modulation in females is less dependent on opioids. If this is the case, then direct activation of PAG output neurons should produce greater antinociception in females, not males. Microinjection of the GABA antagonist bicuculline into the PAG produces antinociception [110, 112] by disinhibiting output neurons. Surprisingly, even though females have more output neurons, the antinociceptive effect of microinjecting bicuculline into the PAG is greater in males [113].

## 5. Sex Differences in the Development of Tolerance to Morphine

Repeated or continuous administration of morphine into the ventrolateral PAG of male rats has been shown to result in the development of tolerance [26, 114–118]. In addition, blocking opioid binding sites in the ventrolateral PAG attenuates the development of tolerance to systemically administered morphine [26]. Tolerance appears to be mediated by a reduction in MOR signaling efficacy in PAG neurons [119], an effect that is reversed when MOR coupling is enhanced via upregulated adenylate cyclase activity [120]. If the PAG-RVM pathway is essential for the development of tolerance, then activation of the PAG-RVM pathway by morphine should decline as tolerance develops, and changes in the activation of this pathway would correlate with sex differences in the development of tolerance to morphine. These hypotheses were tested in male and female rats using behavioral testing (hot plate) and immunohistochemistry to map the activation of the PAG-RVM pathway following repeated morphine administration.

**Figure 5:**
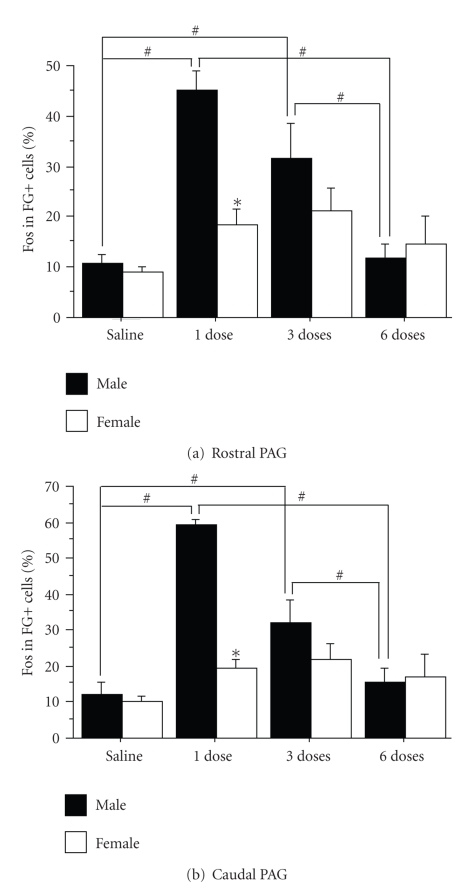
Percentage of Fos-positive neurons that were retrogradely labeled from the RVM (%Fos in FG+ cells) in male (solid bars) and female (open bars) rats injected with either morphine or saline once or twice daily for three days for the rostral ((a); Bregma −6.72, −7.04, −7.74) and caudal ((b); Bregma −8.00, −8.30, −8.80) PAG. A decrease in labeling is evident with an increase in the number of morphine injections for male rats. The *#* indicates a significant effect of treatment and the ∗ indicates a significant effect of sex. *Saline: morphine naïve; 1 dose: saline pretreatment followed by one dose of morphine; 3 doses: one dose of morphine per day; 6 doses: two doses of morphine per day.*

Morphine was administered once or twice a day for three days in rats that had previously received retrograde tracer injections into the RVM. To examine the activation of PAG-RVM neurons during the development of tolerance, males and females were both administered 5 mg/kg of morphine, the ED_50_ for males. Repeated administration of systemic morphine induced tolerance in males to a significantly greater extent than in females [83], consistent with previous research administering equipotent doses of morphine to examine sex differences in tolerance [47]. The half maximal antinociceptive effect of a single injection of morphine following the development of morphine tolerance was two times greater for female compared to male rats. In parallel, the activation of PAG-RVM neurons was significantly attenuated following repeated morphine administration in males [83]. While there was no sex difference in the activation of the PAG following three doses or six doses of morphine over three days (see the black circles in Figures 4(d)–4(i)), the activation of the PAG-RVM projection neurons steadily declined in males only (see the stars in Figures 4(d)–4(i)). Activation of the PAG-RVM pathway by morphine in female rats was minimal, and therefore did not decline significantly following repeated administration of morphine (Figure 5; previously published [83]).

While together, these data provide compelling support for a central role of the PAG in the development of morphine tolerance; these studies administered the *male* ED_50_ dose of morphine. While a single administration of this dose of morphine resulted in comparable activation of the PAG in males and females, it was suboptimal in producing behaviorally defined antinociception in females and may account for why females did not develop tolerance to the same degree as males. Future studies employing sex-specific ED_50_ doses are clearly warranted.

## 6. Role of Gonadal Hormones in Sex Differences in Morphine Analgesia

Studies in rodents indicate that sex differences in the organizational and activational effects of the gonadal hormones estradiol and testosterone influence morphine analgesia. For example, male rats castrated at birth demonstrate decreased morphine potency in adulthood, while female rats masculinized at birth demonstrate greater morphine potency in adulthood [121, 122]. Similarly, morphine is less effective in gonadectomized adult males and is more effective in ovariectomized adult females [40, 123–128]; these effects can be reversed with hormone replacement [44, 123, 129]. Moreover, the antinociceptive potency of morphine has been reported to be greater during diestrus, when circulating estradiol levels are lowest [43, 124, 125, 127, 130], which is corroborated by our recent findings that MOR expression in female rats is the highest during diestrus compared to proestrus and estrus [38]. Recently, it was reported that microinjection of morphine directly into the PAG produces less antinociception during estrus (after estradiol peaks), while there was no sex difference in morphine potency between diestrus females and males [131]. We have recently reported similar findings in which the antihyperalgesic effects of intra-PAG morphine were significantly greater in females in diestrus in comparison to proestrus and estrus [38]. 

The anatomical substrate(s) whereby gonadal steroids influence pain and analgesia is unknown. Both androgen (AR) and estrogen receptors*α* (ER*α*) have been localized in the PAG in the male rat [132]. Although it is not known if these receptors are present in the female rat, they have been localized in other species including the female cat [12], golden hamster [133], guinea pig [134], and the rhesus monkey [135, 136]. To date, however, the anatomical distribution of both types of steroid receptor within the PAG in reference to cells projecting to the RVM is not known.

We have combined neuroanatomical tract-tracing techniques and steroid receptor immunohistochemistry to characterize the expression of AR and ER*α* in the PAG-RVM pathway of male and female rats [137]. In these studies, we found that males had a significantly greater number of AR immunoreactive neurons localized within the dorsomedial, lateral and ventrolateral PAG compared to females. Interestingly, both the qualitative and quantitative expression of ER*α* in the PAG was comparable between the sexes (see Figures 2(d)–2(f)). Both receptor types were preferentially localized within the dorsomedial, lateral and ventrolateral subdivisions of the PAG and increased in density along the rostrocaudal axis of the PAG with the highest expression localized within the caudal PAG. In addition, 30–37% of PAG-RVM output neurons expressed AR or ER*α* (Figure 3(d)) with the highest density of colabeling in the lateral/ventrolateral region of PAG. ERa and AR colocalization in PAG neurons projecting to the RVM was comparable between the sexes [137] (Figures 2(g)–2(i)). The high density of steroid receptors localized on PAG-RVM output neurons may contribute to our observed sex differences in morphine analgesia. Although there was no sex difference in the anatomical localization of gonadal steroid receptors in the PAG despite the higher density of AR in males, 27–50% of PAG-RVM neurons contain MOR [37]. Given that morphine activates more of these neurons in male compared to female rats, the interaction between morphine and sex hormones is likely greater in the PAG of male compared to female rats.

There are several mechanisms whereby gonadal steroids may modulate opioid-sensitive PAG-RVM output neurons, thereby potentially resulting in a dimorphic response to morphine. First, estradiol has been shown to uncouple the MOR from G protein-gated inwardly rectifying potassium channels [138] resulting in an attenuation of morphine-induced hyperpolarization. Second, estradiol has also been shown to induce MOR internalization [139], thereby reducing available opioid binding sites on the cell membrane. Interestingly, ER*α* is required for estradiol-induced MOR internalization [140] supporting the hypothesis that colocalization of MOR and ER*α* in the PAG-RVM output neurons may provide a pain modulatory mechanism. Interestingly, administration of estradiol to gonadectomized males reinstates morphine analgesia while dihydrotestosterone does not [141], suggesting that estrogens affect morphine potency in both male and female rats [130].

## 7. Conclusions

Research spanning for four decades has shown that the PAG and its descending projections to the RVM and spinal cord dorsal horn constitute an essential neural circuit for opioid-based analgesia. During the last half of that period, numerous rodent and human studies have established sex differences in the antinociceptive and analgesic effects of morphine; however, the neural mechanisms underlying the sexually dimorphic actions of morphine remain poorly understood. It is now clear that the anatomical and physiological characteristics of the PAG and its descending projections to the RVM are sexually dimorphic, with clear biological consequences in terms of morphine potency. Our studies, as well as those of others, have shown that morphine is less potent in females compared to males in the alleviation of persistent pain. Future research efforts utilizing female subjects in both the investigation of persistent pain mechanisms and identification of both effective and potent pain therapeutics are clearly warranted.
